# Subclassification of epithelioid sarcoma with potential therapeutic impact

**DOI:** 10.1002/path.6135

**Published:** 2023-06-14

**Authors:** Simon Haefliger, Olga Chervova, Christopher Davies, Steven Nottley, Steven Hargreaves, Vaiyapuri P Sumathi, Fernanda Amary, Roberto Tirabosco, Nischalan Pillay, Stephan Beck, Adrienne M Flanagan, Iben Lyskjær

**Affiliations:** ^1^ Research Department of Pathology University College London, UCL Cancer Institute London UK; ^2^ Institute of Medical Genetics and Pathology University Hospital Basel Basel Switzerland; ^3^ Department of Histopathology Royal National Orthopaedic Hospital Stanmore UK; ^4^ Medical Genomics Research Group University College London, UCL Cancer Institute London UK; ^5^ The Royal Orthopaedic Hospital Birmingham UK; ^6^ Department of Molecular Medicine Aarhus University Hospital Aarhus Denmark

**Keywords:** DNA methylation profile, epithelioid sarcoma, *SMARCB1*, immune cell deconvolution, *SMARCB1*‐deficient tumours

## Abstract

Epithelioid sarcoma is a rare and aggressive mesenchymal tumour, the genetic hallmark of which is the loss of expression of *SMARCB1*, a key member of the SWItch/Sucrose Non‐Fermentable (SWI/SNF) chromatin remodelling complex. Hampered by its rarity, epithelioid sarcoma has received little research attention and therapeutic options for this disease remain limited. *SMARCB1*‐deficient tumours also include malignant rhabdoid tumour, atypical teratoid and rhabdoid tumour, epithelioid malignant peripheral nerve sheath tumour, and poorly differentiated chordoma. Histologically, it can be challenging to distinguish epithelioid sarcoma from malignant rhabdoid tumour and other *SMARCB1‐*deficient tumours, whereas methylation profiling shows that they represent distinct entities and facilitates their classification. Methylation studies on *SMARCB1*‐deficient tumours, although not including epithelioid sarcomas, reported methylation subgroups which resulted in new clinical stratification and therapeutic approaches. In addition, emerging evidence indicates that immunotherapy, including immune checkpoint inhibitors, represents a promising therapeutic strategy for *SMARCB1‐*deficient tumours. Here, we show that some epithelioid sarcomas share methylation patterns of malignant rhabdoid tumours indicating that this could help to distinguish these entities and guide treatment. Using gene expression data, we also showed that the immune environment of epithelioid sarcoma is characterised by a predominance of CD8^+^ lymphocytes and M2 macrophages. These findings have potential implications for the management of patients with epithelioid sarcoma. © 2023 The Authors. *The Journal of Pathology* published by John Wiley & Sons Ltd on behalf of The Pathological Society of Great Britain and Ireland.

## Introduction

Epithelioid sarcoma (EpS) is an aggressive mesenchymal tumour, characterised by the loss of expression of *SMARCB1*, a key member of the SWItch/Sucrose Non‐Fermentable (SWI/SNF) chromatin remodelling complex. EpS presents most commonly in young adults (20–40 years old) and is divided into two clinico‐morphological subtypes: the classical (or peripheral) EpS occurring mainly in the extremities and the proximal (or central) EpS arising in the truncal region of the body [1]. Hampered by its rarity, EpS has been poorly studied, resulting in limited treatment options. Recent clinical trials identified the *EZH2* inhibitor tazemetostat as a promising treatment for EpS, with 15% of patients showing a response (reduction in tumour size) [2]. More recently, a publication from a multi‐institutional consortium using whole exome and whole transcriptome data provided new insight into EpS and revealed biological differences between the two EpS subtypes, paving the way for potential targeted treatment [3].

The loss of the SMARCB1 protein expression, in addition to occurring in EpS, is also a hallmark of other neoplasms, notably of some rare paediatric tumours, including malignant rhabdoid tumour (MRT) and atypical teratoid and rhabdoid tumour (ATRT), both of which have a poor prognosis and arise in children with a mean age of diagnosis of 1–2 years [4, 5]. Epithelioid malignant peripheral nerve sheath tumour (eMPNST) and poorly differentiated chordoma (PDC) represent other *SMARCB1‐*deficient tumours. Histologically, these entities share features, and it can be challenging to distinguish between them. Genome‐wide methylation studies have advanced the classification of these tumours and revealed that they form separate clusters [6]. Furthermore, subgroups of ATRT including ATRT‐MYC, ATRT‐TYR, and ATRT‐SHH have been identified. ATRT‐MYC is associated with MYC overexpression; ATRT‐TYR is characterised by overexpression of melanosomal marker genes, particularly tyrosinase; and ATRT‐SHH exhibits high Sonic Hedgehog and NOTCH signalling [5]. These studies resulted in new clinical therapeutic approaches [5]. It is noteworthy that genome‐wide methylation studies focusing specifically on EpS have not been produced so far.

There is emerging evidence that genetic alterations in the SWI/SNF complex and its core subunits can act as biomarkers for predicting treatment response to immune checkpoint inhibitors (ICIs) [7]. Indeed, MRTs have recently been reported as being immunogenic, despite their non‐complex genome and low tumour mutational burden [8]. This led to treatment of *SMARCB1*‐deficient tumours with ICIs as monotherapy, dual therapy, or in combination with other anti‐tumour agents in clinical trials; these included patients with EpS, although the numbers were limited [9].

Here, we explored the methylation landscape and the immune microenvironment of EpS in the context of other *SMARCB1‐*deficient tumours [10], with the aim of identifying potential therapeutic targets.

## Materials and methods

### Patient samples

Patient tissues and data were obtained from the Royal National Orthopaedic Hospital (Stanmore, UK) and the Royal Orthopaedic Hospital (Birmingham, UK), which are covered by the Human Tissue Authority licences 12055. Ethical approval was obtained from the Cambridgeshire 2 Research Ethics Service (reference 09/H0308/165‐2020).

### Sample selection

The pathology archives were searched for sarcomas ICD‐coded as ‘epithelioid sarcoma’. Twenty‐five epithelioid sarcomas (16 classical EpSs, nine proximal EpSs) were included in this study. All cases were reviewed by expert pathologists (AMF and RT), who selected cases that fulfilled the WHO diagnostic criteria [11] for EpS. All cases showed loss of expression of *SMARCB1* by immunohistochemistry.

Supplementary material, Table S1 and Figures S1 and S2 provide demographic, clinical, and morphological data for the cohort.

### Publicly available data

Publicly available transcriptomic data from the TARGET database [12], MRTs (*n* = 40), and GSE70678 [5], ATRTs (*n* = 49), were used for analysis, along with methylation data from GSE70460 [5], ATRTs (*n* = 150), and GSE140686 [6], MRTs (*n* = 17). An additional 18 cases of EpS from the reference dataset of the DKFZ sarcoma classifier, GSE140686 [6], were used for validation. From this dataset, we also included other sarcomas for validation purposes. These included fusion‐driven sarcomas [Ewing sarcoma (EWS) (*n* = 35), synovial sarcoma (SYSA) (*n* = 39), dermatofibrosarcoma protuberans (DFSP) (*n* = 37)] and ‘complex’ sarcomas [undifferentiated sarcoma (UPS) (*n* = 22) and leiomyosarcoma (LMS) (*n* = 16)].

### DNA and RNA extraction for fresh‐frozen (FF) and formalin‐fixed, paraffin‐embedded (FFPE) samples

DNA was extracted from 25 tumour samples, of which 16 samples (FF) were used for methylation analysis. Eighteen EpS tumour samples, nine FFPE and nine FF, underwent RNA extraction (Supplementary materials and methods and Table S1).

### RNA sequencing protocol

Raw reads from fastq files were aligned to the GRCh38 build of the human reference genome using HISAT2, and gene expression was quantified using featureCounts (Supplementary materials and methods).

### DNA methylation protocol and processing of data

Aliquots (500 ng) of DNA were bisulphite‐converted using a Zymo EZ DNA Methylation‐Gold Kit (Zymo Research Corporation, Irvine, CA, USA) and hybridised to Infinium HumanMethylationEPIC BeadChip arrays (Illumina, San Diego, CA, USA) according to the manufacturer's recommendations. The generated IDAT files were processed using the ChAMP R‐package (Supplementary materials and methods).

### Methylome profile analysis

The 10,000 most variable CpGs were used to perform hierarchical clustering (ward.D2 method) and dimension reduction using the uniform manifold approximation and projection (UMAP) method [13] (Supplementary materials and methods).

### Analysis through the DKFZ sarcoma classifier

The raw methylation files (IDAT files) of the 16 EpS samples were run through the DKFZ sarcoma classifier [14] (supplementary material, Table S2).

### Immune cell deconvolution

To deconvolute gene expression signals originating from 22 immune cell types, we applied the CIBERSORT algorithm [14] to infer the relative proportions of immune cell types to 18 EpS, 40 MRT, and 49 ATRT samples.

### Statistical analysis

To compare the inferred immune scores between tumour types, a two‐sided *t*‐test with significance threshold *p* = 0.05 was applied. To account for multiple testing, we used the Bonferroni correction. For correlation analysis, we calculated the Pearson and Spearman correlation coefficient rho (rPear, rSpear). Statistical analysis and data plotting were performed using R version 4.2 (R Foundation for Statistical Computing, Vienna, Austria) using the following packages: ggplot2, ggbpur, uwot, and ComplexHeatmap.

## Results

### Methylation analysis of EpS across *SMARCB1* ‐deficient neoplasms reveals specific clusters

**Figure 1:**
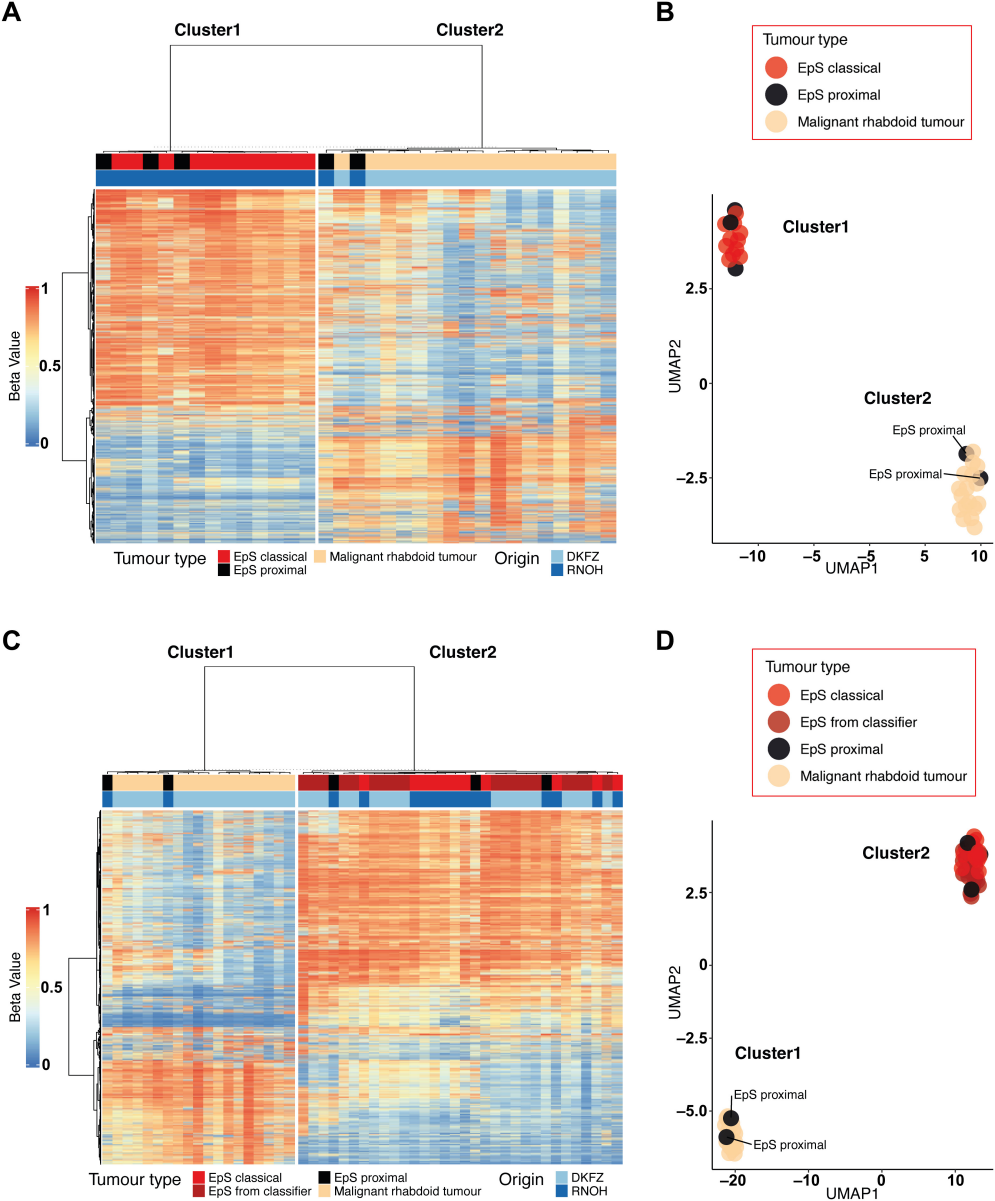
Hierarchical clustering and dimension reduction in EpS and MRT. (A, B) Heatmap displaying hierarchical clustering and UMAP analysis including EpS (*n* = 16) and MRT (*n* = 17) samples. Two EpS samples of the proximal type cluster together with the MRT samples. (C, D) Heatmap displaying hierarchical clustering (C) and UMAP analysis (D) including EpS (*n* = 16), MRT (*n* = 17), and additional EpS samples from the DKFZ sarcoma classifier (*n* = 18). Two EpS samples of the proximal type cluster together with the MRT samples. See the Materials and methods section for details of publicly available data.

**Figure 2:**
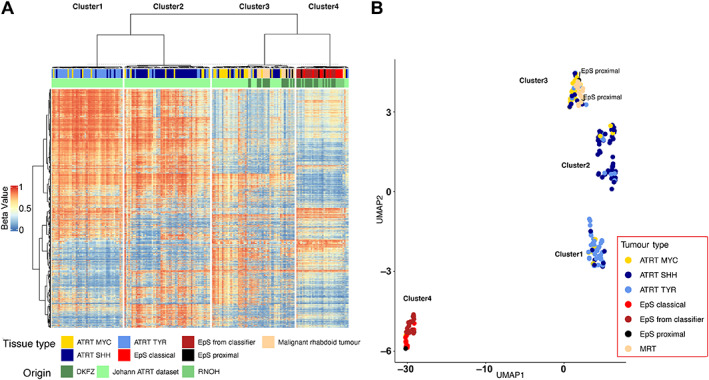
Hierarchical clustering and dimension reduction of *SMARCB1*‐deficient neoplasms. (A, B) Heatmap displaying hierarchical clustering and UMAP analysis including EpS (*n* = 16), EpS from the DKFZ classifier (*n* = 18), MRT (*n* = 17), and ATRT (*n* = 150) samples. Four clusters across *SMARCB1*‐deficient neoplasms are delineated: cluster1 (ATRT‐TYR), cluster2 (ATRT‐SHH), cluster3 (ATRT‐MYC and MRT), and cluster4 (EpS). Two EpS samples of the proximal type cluster together with the MRT samples. See the Materials and methods section for details of publicly available data.

**Figure 3:**
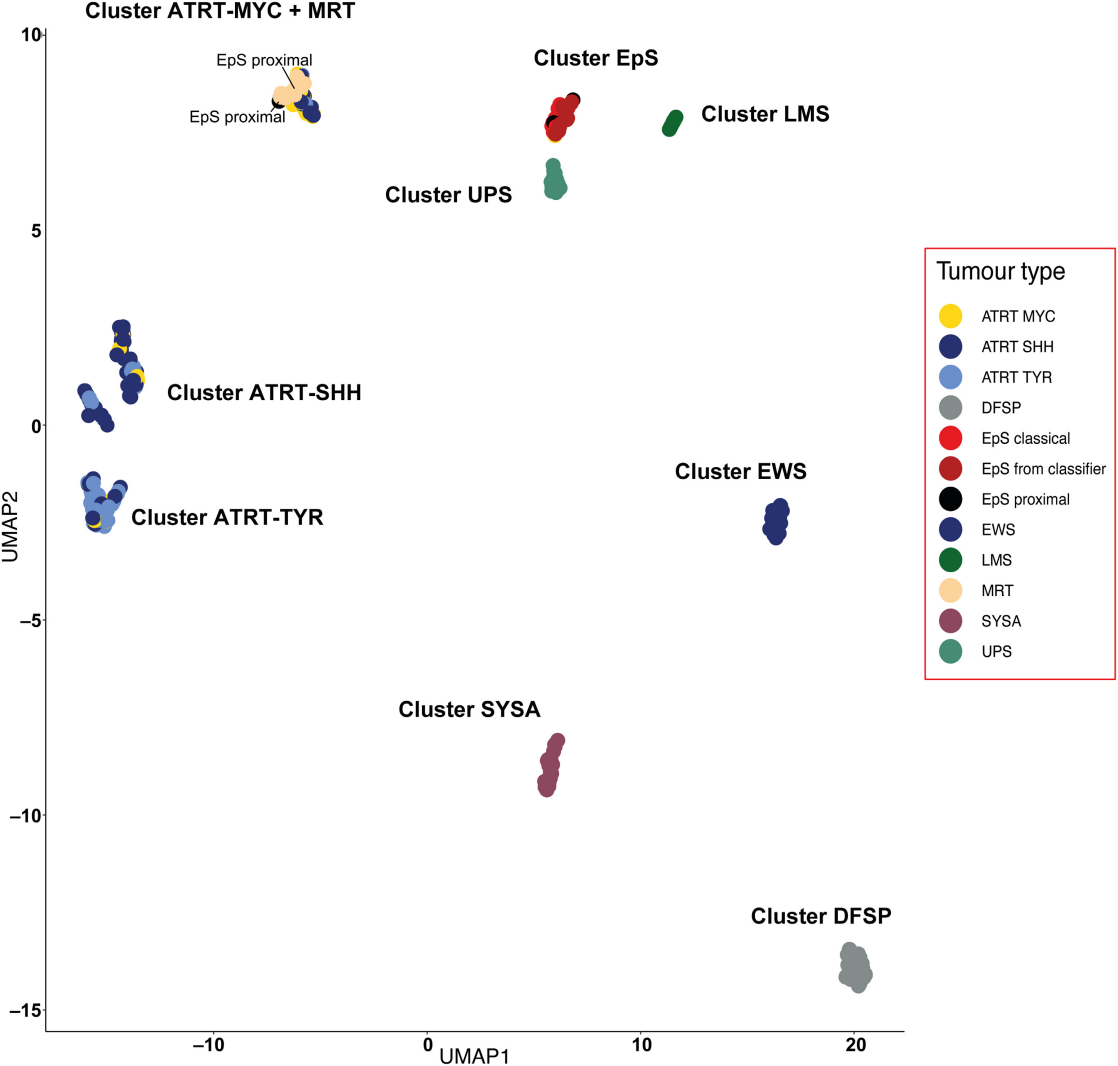
UMAP analysis of *SMARCB1*‐deficient neoplasms across sarcomas. UMAP analysis including EpS (*n* = 16), MRT (*n* = 17), ATRT (*n* = 150), EpS from the DKFZ classifier (*n* = 18), Ewing sarcoma (EWS, DKFZ) (*n* = 35), synovial sarcoma (SYSA, DKFZ) (*n* = 39), dermatofibrosarcoma protuberans (DFSP, DKFZ) (*n* = 37), class undifferentiated sarcoma (UPS, DKFZ) (*n* = 22), and leiomyosarcoma (LMS, DKFZ) (*n* = 16). The four clusters found across *SMARCB1*‐deficient neoplasms [cluster1 (ATRT‐TYR), cluster2 (ATRT‐SHH), cluster3 (ATRT‐MYC and MRT), and cluster4 (EpS)] could also be delineated when extending the analysis to additional sarcoma subtypes. See the Materials and methods section for details of publicly available data.

Cluster analysis comparing EpS with MRT revealed that two of five proximal‐type EpSs showed a methylation pattern similar to that of MRT (Figure 1A,B). This result was confirmed by interrogating the samples in an external dataset of additional EpSs (Figure 1C,D and supplementary material, Figures S3 and S4). We then performed cluster analysis of EpS, MRT, and ATRT (Figure 2A,B and supplementary material, Figure S5) and delineated four clusters across *SMARCB1*‐deficient neoplasms: cluster1 (ATRT‐TYR), cluster2 (ATRT‐SSH), cluster3 (ATRT‐MYC and MRT), and cluster4 (EpS). These four clusters were validated using additional sarcoma samples of different subtypes (Figure 3). In each of the analyses, two of five proximal‐type EpSs showed a methylation pattern similar to that of MRT (Figure 2A,B, Figure 3, and supplementary material, Figure S6). These results are consistent with the previously reported methylation subgroups of ATRT and MRT [5, 15].

### Immune cell deconvolution shows a predominance of M2 macrophages and CD8 ^+^ lymphocytes in the EpS tumour microenvironment (TME)

**Figure 4:**
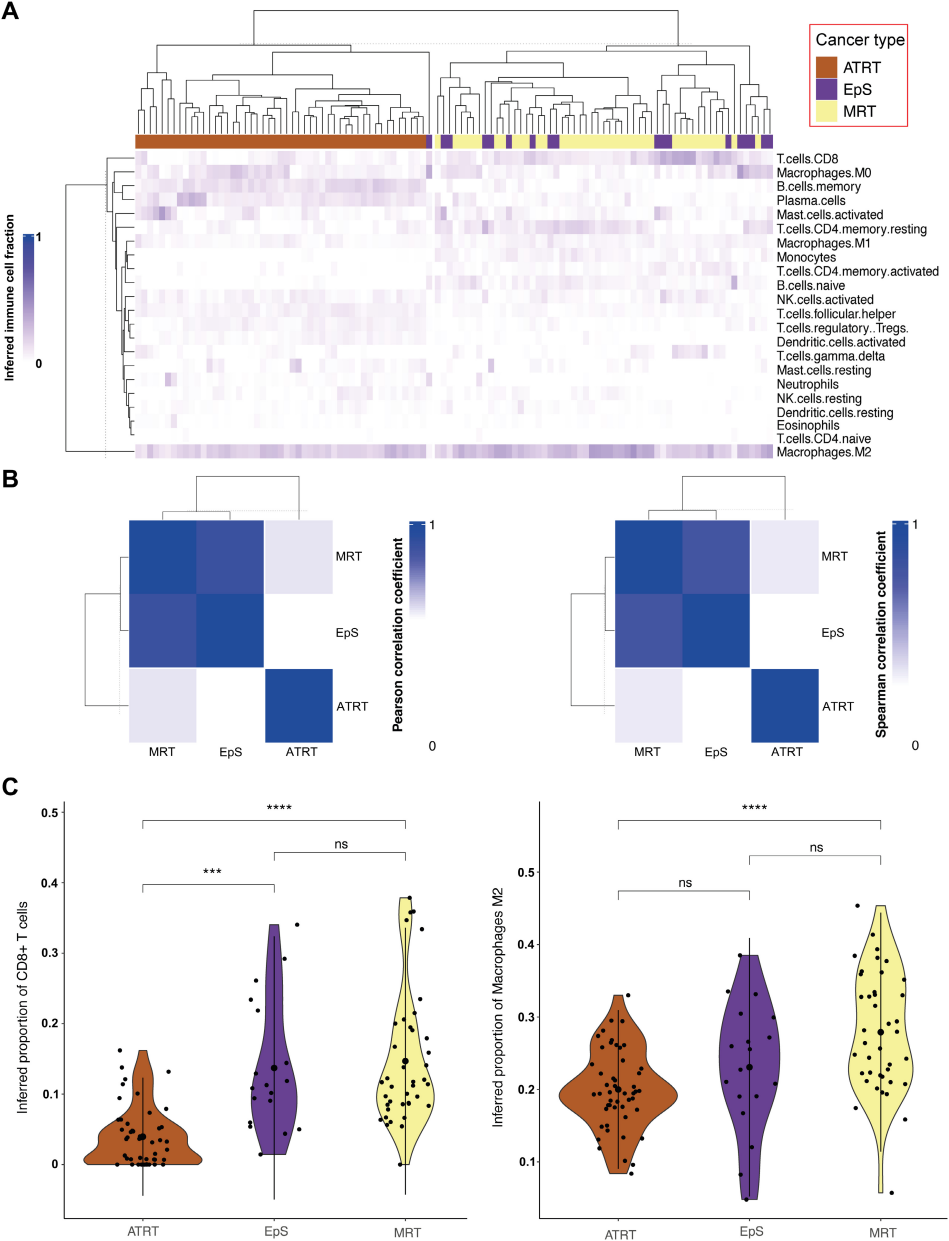
Summary of *in silico* immune cell deconvolution in *SMARCB1*‐deficient neoplasms, EpS, MRT, and ATRT. (A) Heatmap displaying hierarchical cluster based on the percentage of inferred immune cell type per sample for EpS (*n* = 18), MRT (*n* = 40), and ATRT (*n* = 49). (B) Correlation plot (rPear left, rSpear right) based on the mean value of each inferred immune cell type across *SMARCB1*‐deficient neoplasms. (C) Violin plots showing the inferred fraction of CD8^+^ T cells (left) and macrophages M2 (right) in EpS, MRT, and ATRT. See the Materials and methods section for details of publicly available data.

Correlating all the inferred relative proportions of the 22 immune cell types used for deconvolution across EpS, MRT, and ATRT, we observed that EpS (combining the proximal and classical EpS cases) and MRT correlated most closely (rPear = 0.9, rSpear = 0.8) (Figure 4A,B). A weaker correlation was observed between EpS and ATRT (rPear = 0.57, rSpear = 0.33), and between MRT and ATRT (rPear = 0.61, rSpear = 0.26). The CD8^+^ cytotoxic T cells and M2 macrophages showed the highest inferred relative proportion amongst the 22 deconvoluted immune cells in EpS (supplementary material, Figure S7). No significant differences were identified in the inferred proportion of immune cells between the classical and proximal types of EpS (supplementary material, Figure S8). Comparing the inferred immune cell types across EpS, MRT, and ATRT, we observed similar proportions of CD8^+^ cytotoxic T cells between EpS and MRT (*p* = 0.96), whereas a lower proportion were detected in ATRT (*p* < 0.05) (Figure 4C). M2 macrophages showed the highest inferred fraction in each of the three tumours (Figure 4C). Supplementary material, Figure S9 provides an overview of the relative proportion of all immune cells inferred across samples.

## Discussion

Here, we provide new insights into the methylation landscape and immune environment of EpS. We identified two cases of proximal EpS which exhibit a methylation profile similar to that of MRT, a tumour occurring at a much younger age. Using the DKFZ sarcoma classifier, we were able to extend our analysis to other types of sarcomas and validate our findings.

The methylation clustering together of some proximal EpSs and MRTs is noteworthy as others have previously reported that there are significant morphological similarities between these two entities [16, 17]. In some cases, pathologists find it difficult to distinguish MRT, EpS, and poorly differentiated chordoma purely on haematoxylin and eosin staining. Whereas expression of brachyury provides with certainty a diagnosis of poorly differentiated chordoma, there are no specific biomarkers to distinguish MRT and EpS. By convention, distinguishing MRT from EpS is based on age at presentation. MRTs present in infants, whereas this is rarely the case for EpSs. Our findings show the benefit of methylation profiling of *SMARCB1*‐deficient tumours and in particular its value in separating MRT from EpS. This is important as clinical management for these tumours is different [18, 19]. While treatment is mainly surgical for EpS, most MRT patients are treated with intensive multimodal regimens, combining early surgical resection of primary tumour, intensive multidrug chemotherapy regimen, and local radiotherapy to all sites of disease involvement. Furthermore, our finding could also play an important role in the selection of patients for relevant targeted treatments and clinical trials. As methylome profiling can be used in a routine clinical setting for classifying a variety of tumours, it could be introduced with relative ease into clinical practice and thereby provide a more robust diagnosis of EpS than is currently available.

Finally, our study of the TME in EpS supports a growing body of evidence derived from case reports and clinical trials that EpS should be considered an immunogenic neoplasm and may benefit from ICI treatment [9].

The limitation of our work is the small number of cases available for analysis, which is inherent to the study of rare neoplasms. However, we hope that this short report will prompt others to undertake methylation profiling on *SMARCB1‐*deficient tumours, as the classification of disease has important implications for treatment. This would also demonstrate whether methylation profiling proves to be a useful diagnostic adjunct, particularly in cases where a diagnosis on histology alone is challenging. Furthermore, an independent validation cohort may demonstrate whether EpSs with MRT features also behave clinically like MRTs.

## Author contributions statement

AMF, SH and IL conceptualised the study. CD and SH curated the data. SH, AMF, IL and OC carried out the formal analysis. AMF, SH and IL acquired funding. SH, OC, CD, SN, SH, VPS, FA, RT, NP, SB, AMF and IL conducted the investigation. SH, OC, AMF and IL wrote the original draft. All authors made a contribution to the final manuscript.

## Supporting information


Supplementary materials and methods
**Figure S1.** Representative histology of the two EpS cases (S00066445, S00097208) clustering with MRT showing cellular lesions infiltrating the surrounding connective tissue
**Figure S2.** Representative histology of two EpS cases
**Figure S3.** UMAP analysis including EpS (*n* = 16) and MRT (*n* = 17) with different n_neighbours values in the regression model
**Figure S4.** UMAP analysis including EpS (*n* = 16), MRT (*n* = 17), and additional EpS samples from the sarcoma classifier (*n* = 18) with different n_neighbours values in the regression model
**Figure S5.** UMAP analysis including EpS (*n* = 16), EpS from the classifier (*n* = 18), MRT (*n* = 17), and ATRT (*n* = 150) samples with different n_neighbours values in the regression model
**Figure S6.** UMAP analysis including EpS (*n* = 16), MRT (*n* = 17), EpS from the classifier (*n* = 18), Ewing's sarcoma (EWS, DKFZ) (*n* = 35), synovial sarcoma (SYSA, DKFZ) (*n* = 39), dermatofibrosarcoma protuberans (DFSP, DKFZ) (*n* = 37), class undifferentiated sarcoma (UPS, DKFZ) (*n* = 22), and leiomyosarcoma (LMS, DKFZ) (*n* = 16) with different n_neighbours values in the regression model.
**Figure S7.** Boxplots comparing all inferred proportions of immune cells in the two EpS subtypes (*n* = 18)
**Figure S8.** Barplots showing the inferred fraction of the 22 immune cell types included in the CIBERSORT matrix reference in EpS (*n* = 18), MRT (*n* = 40), and ATRT (*n* = 49)
**Figure S9.** Boxplots comparing all inferred proportions of immune cells in *SMARCB1*‐deficient neoplasms, EpS (*n* = 18), MRT (*n* = 40), and ATRT (*n* = 49)

**Table S1.** Summary of the epidemiological, clinical, and morphological features of the EpS cohort

**Table S2.** DKFZ sarcoma classifier results of the EpS cohort

## Data Availability

The data that support the findings of this study are openly available in the European Genome‐Phenome Archive (EGA), Reference Number EGAS00001007257.
