# Supplementary material for: Subclassification of epithelioid sarcoma with potential therapeutic impact
Source: J Pathol. 2023 Jun 14;260(4):368–75. doi: 10.1002/path.6135 (PMC10952852; doi:10.1002/path.6135)
Supplement: Supplementary file 1 — Supplementary materials and methods Figure S1. Representative histology of the two EpS cases (S00066445, S00097208) clustering with MRT showing cellular lesions infiltrating the surrounding connective tissue Figure S2. Representative histology of two EpS cases Figure S3. UMAP analysis including EpS (n = 16) and MRT (n = 17) with different n_neighbours values in the regression model Figure S4. UMAP analysis including EpS (n = 16), MRT (n = 17), and additional EpS samples from the sarcoma classifier (n = 18) with different n_neighbours values in the regression model Figure S5. UMAP analysis including EpS (n = 16), EpS from the classifier (n = 18), MRT (n = 17), and ATRT (n = 150) samples with different n_neighbours values in the regression model Figure S6. UMAP analysis including EpS (n = 16), MRT (n = 17), EpS from the classifier (n = 18), Ewing's sarcoma (EWS, DKFZ) (n = 35), synovial sarcoma (SYSA, DKFZ) (n = 39), dermatofibrosarcoma protuberans (DFSP, DKFZ) (n = 37), class undifferentiated sarcoma (UPS, DKFZ) (n = 22), and leiomyosarcoma (LMS, DKFZ) (n = 16) with different n_neighbours values in the regression model. Figure S7. Boxplots comparing all inferred proportions of immune cells in the two EpS subtypes (n = 18) Figure S8. Barplots showing the inferred fraction of the 22 immune cell types included in the CIBERSORT matrix reference in EpS (n = 18), MRT (n = 40), and ATRT (n = 49) Figure S9. Boxplots comparing all inferred proportions of immune cells in SMARCB1‐deficient neoplasms, EpS (n = 18), MRT (n = 40), and ATRT (n = 49) [file PATH-260-368-s003.docx]

**Subclassification of epithelioid sarcoma with potential therapeutic impact**

S Haefliger *et al. J Pathol* <https://doi.org/10.1002/path.6135>

**Supplementary materials and methods**

**Supplementary Figures S1–S9**

**Supplementary Tables S1 and S2 (supplied as separate Excel files)**

**Supplementary materials and methods**

*DNA and RNA extraction for the fresh-frozen (FF) samples*

Tumour DNA was extracted from 10-μm sections of snap-frozen fresh samples using a QIAamp DNA Mini Kit (Qiagen, Hilden, Germany) using the standard kit protocol and eluted in the kit elution buffer. The samples were incubated overnight, and additional proteinase K was added (up to 20 µl) if the tissue had not fully lysed. All DNA samples were quality-controlled using a NanoDrop™ 1000 (Thermo Fisher Scientific, Waltham MA, USA), Qubit™ dsDNA HS (Thermo Fisher Scientific), and Genomic DNA ScreenTape Assay (Agilent Technologies Inc., Santa Clara, CA, USA). Samples of sufficient purity (A280/260: 1.8–2), concentration (>20 ng/µl), and integrity (DIN score > 6) were sent for analysis. Tumour RNA was extracted from 10-µm sections of the same frozen sample from which the DNA was extracted. The sections were lysed in TRIzol^®^ (Thermo Fisher Scientific) using the Zymo Direct-zol RNA Miniprep Kit (Cambridge Bioscience, Cambridge, UK). The TRIzol^®^ reagent was phase-separated with chloroform, and the aqueous phase run through the standard Zymo Kit protocol with a 15-min DNase step. The RNA was eluted in nuclease-free water. Quality assessment was undertaken using a NanoDrop™ 1000 and RNA ScreenTape Assay (Agilent Technologies Inc.). Samples of sufficient integrity (RIN score > 6) and yield (>1 µg) were submitted for analysis.

*DNA and RNA extraction of formalin-fixed*, *paraffin-embedded* (*FFPE*) *samples*

FFPE tumour RNA was co-extracted with DNA using the truXTRAC FFPE total NA Plus Column Kit (Covaris, Brighton, UK) and Covaris E220 ultrasonicator. Nine 5-µm sections of tumour were added to the kit AFA fibre tubes, sonicated in lysis buffer for 5–7 min, and RNA was extracted using the standard protocol. The samples were digested for 30 min for the RNA extractions, and for a further 2–3 h for the DNA extractions. The RNA was eluted in nuclease-free water and quality-controlled using a NanoDrop™ 1000 and RNA ScreenTape Assay (Agilent Technologies Inc.). RNA samples with a DV200 of above 50% were submitted for analysis.

*Preprocessing of DNA methylation data*

The generated IDAT files were processed using the following *ChAMP* R-package [20] filtering parameters: probes with a detection *p* value above 0.01, probes with < 3 beads in at least 5% of samples per probe, non-CpG probes, all SNP-related probes, multi-hit probes, and probes located in chromosomes X and Y. For assessment of data quality, the champ.QC function was used. Four samples (IDs S00056154, S00056147, S00056153, S00065391) did not pass the quality assessment and were excluded from further analysis. BMIQ was used as a normalisation method. The EPIC array data were converted to a virtual 450K array for joint normalisation and processing of data from both platforms using the combineArrays function in the R-package *minfi* [21]*.* Batch effect was assessed using the singular value decomposition method. Batch effects related to the source of the data, array type (450K versus 850K), and slides were present. The covariates were overlapping with the phenotype of interest (cancer type) as data from different sources with specific characteristics (array type and slides) were combined. No batch effect correction was performed.

*UMAP analysis*

UMAP analysis was performed using the function R-package *uwot* (https://github.com/jlmelville/uwot). For high-dimensional datasets, using principal component analysis (PCA) to reduce dimensionality is recommended by the author of the package. The initial settings used to generate the non-linear regression model were as follows: n_neighbours = 15, input = 10,000 most variable CpGs, and PCA = set to capture 80% of the variance of each dataset (specific value depending on the dataset). To test the robustness of the UMAP analysis, we also performed the analysis by modifying the n_neighbours parameter (8, 15, 25) and obtained similar results (see supplementary material, Table S2 and Figures S3 and S4).

*Immune cell deconvolution*

The following immune cell types were included in the analysis: M0, M1, and M2 macrophages; T follicular helper cells; resting memory CD4 T cells; activated memory CD4 T cells; γδ T cells; CD8 T cells; regulatory T cells; naive CD4 T cells; resting NK cells; activated NK cells; resting mast cells; activated mast cells; memory B cells; resting dendritic cells; activated dendritic cells; naive B cells; monocytes; neutrophils; eosinophils; and plasma cells. Gene-level transcripts per million (TPM) was used for the EpS and MRT cohorts; gene-level derived from the Affymetrix Human Genome U133 Plus 2.0 Arrays platform was used for the ATRT cohort; 5,000 permutations using the absolute signature score mode were used for deconvolution. CIBERSORT derives a *p* value for the deconvolution for each sample using Monte Carlo sampling, providing a measure of confidence in the results. Only results with a value of *p* < 0.05 were considered eligible for further analysis.

**Supplementary Figures S1–S9**


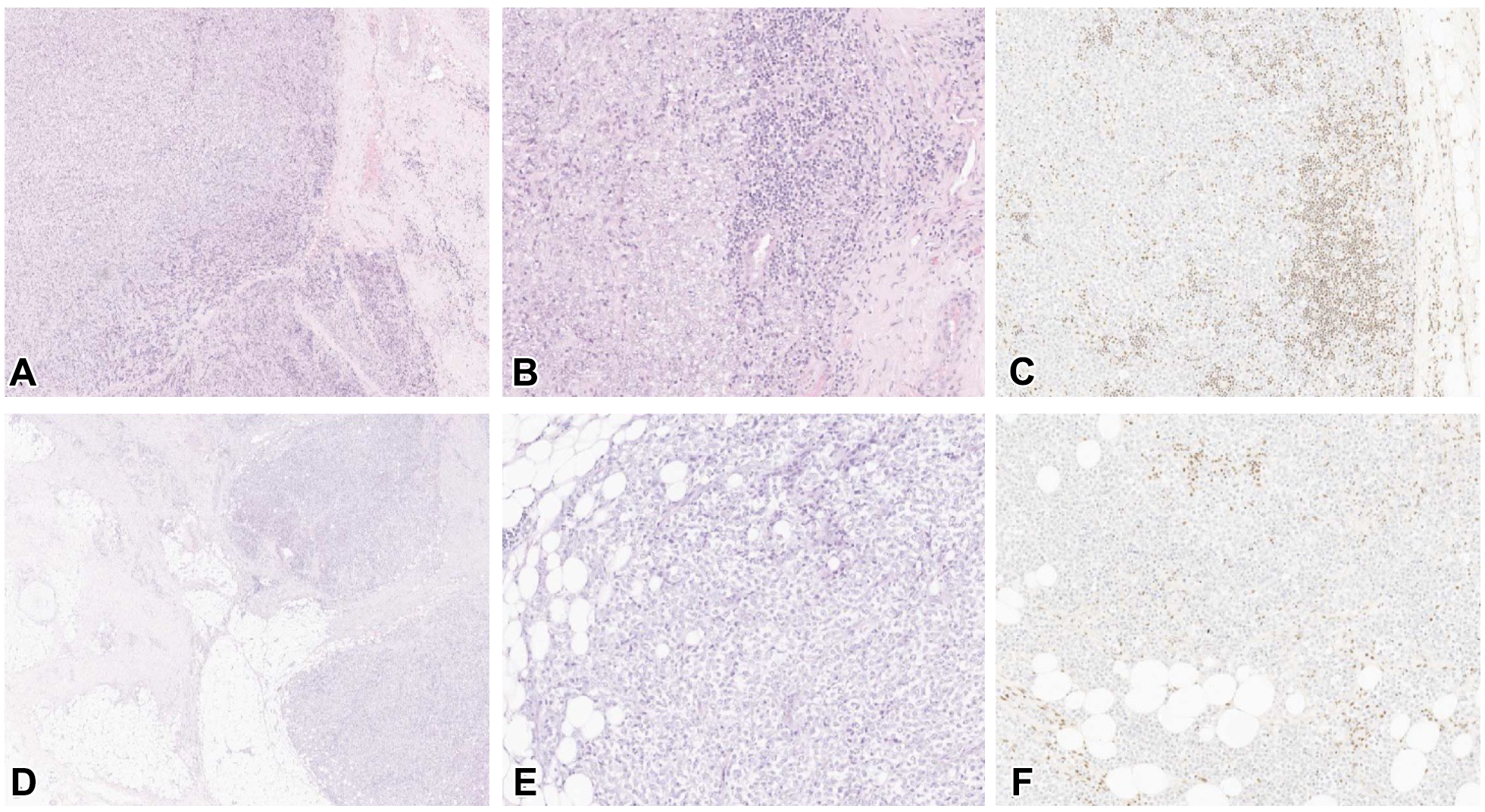


**Figure S1.** Representative histology of the two EpS cases (S00066445, S00097208) clustering with MRT showing cellular lesions infiltrating the surrounding connective tissue (A-B for S00066445, D-E for S00097208). The tumour cells show loss of SMARCB1 expression by immunohistochemistry (C, F), with surrounding lymphocytes acting as a positive control (SMARCB1 expression retained). A-C


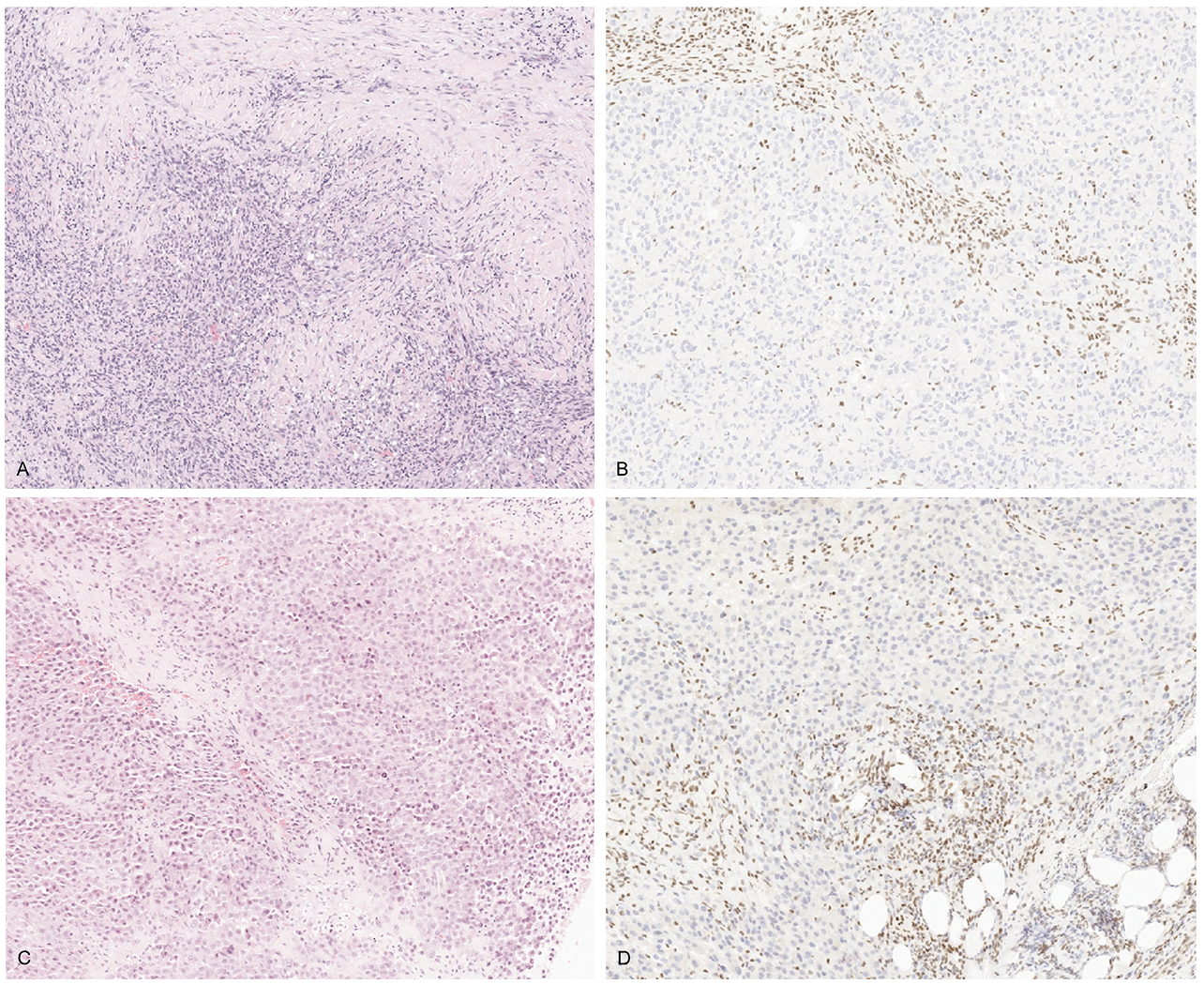


**Figure S2.** Representative histology of two EpS cases. (A) A proximal type (S00067405) and (C) a classical type (S00097179). The tumour cells show loss of SMARCB1 expression by immunohistochemistry (B, D), with surrounding lymphocytes acting as a positive control.


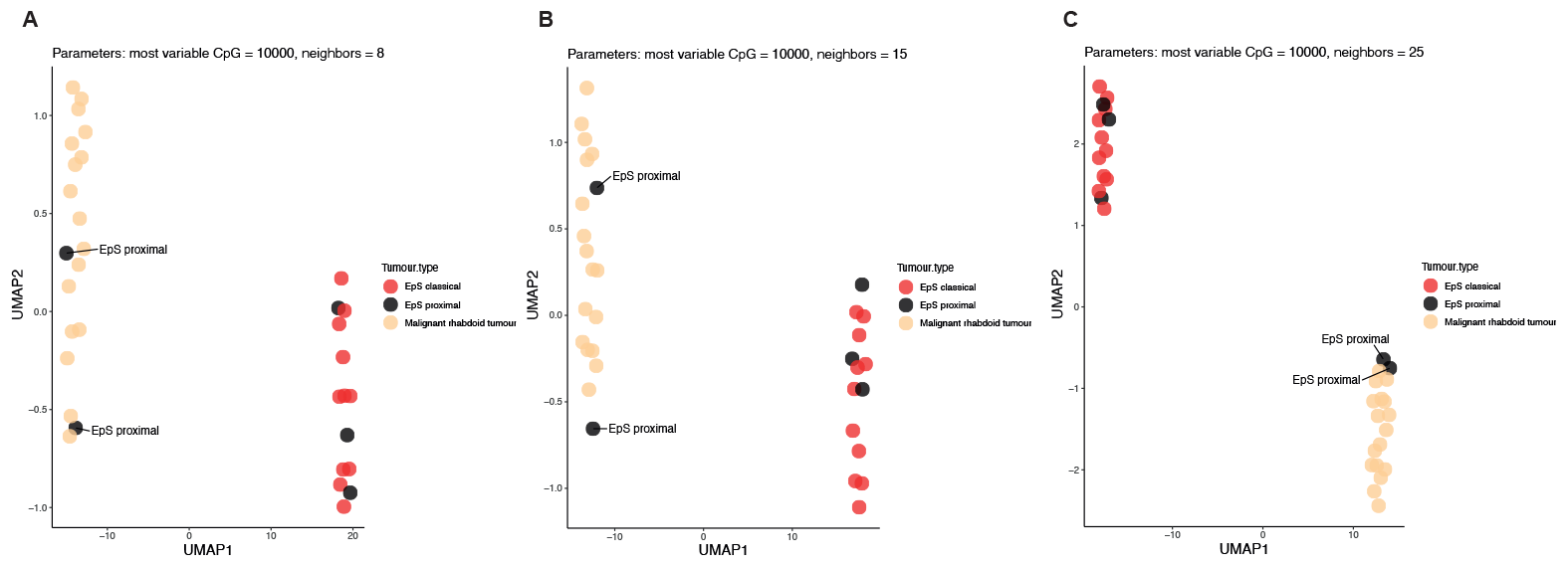


**Figure S3.** UMAP analysis including EpS (*n*= 16) and MRT (*n*= 17) with different n_neighbours values, *n*= 8 (A), *n*= 15 (B), and *n*= 25 (C), in the regression model. Two EpS samples of the proximal type cluster together with the MRT samples.


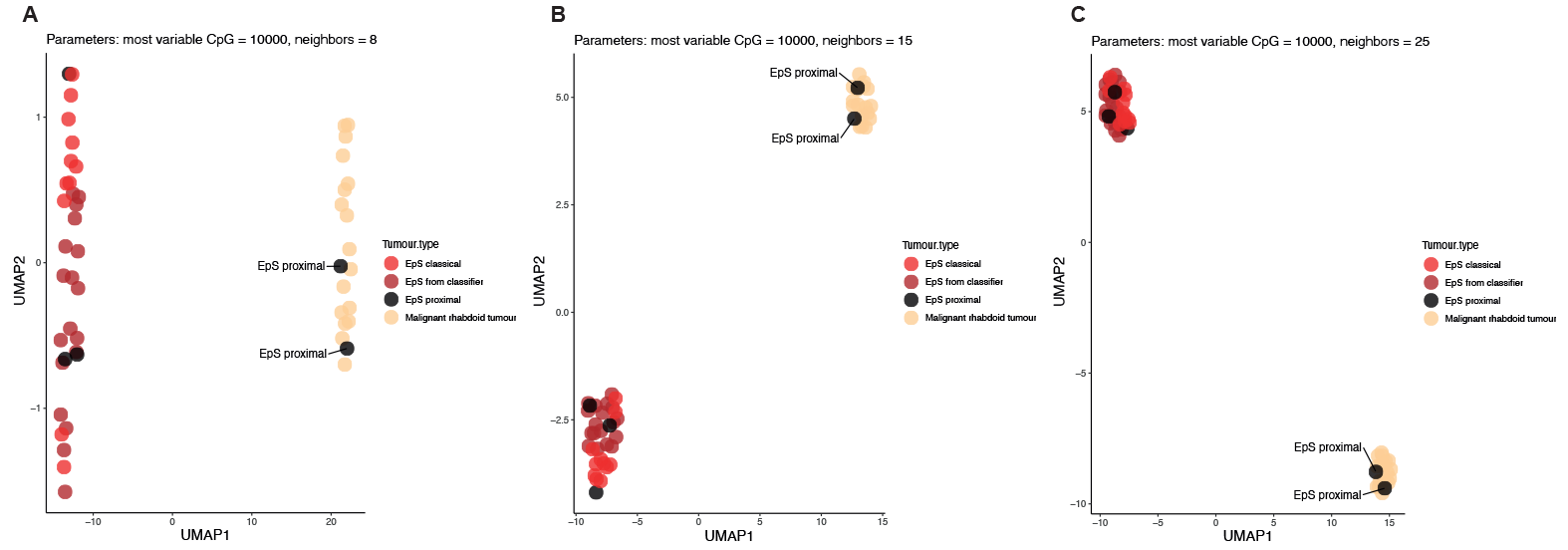


**Figure S4.** UMAP analysis including EpS (*n*= 16), MRT (*n*= 17), and additional EpS samples from the sarcoma classifier (*n*= 18) with different n_neighbours values, *n*= 8 (A), *n*= 15 (B), and *n*= 25 (C), in the regression model. Two EpS samples of the proximal type cluster together with the MRT samples.

**
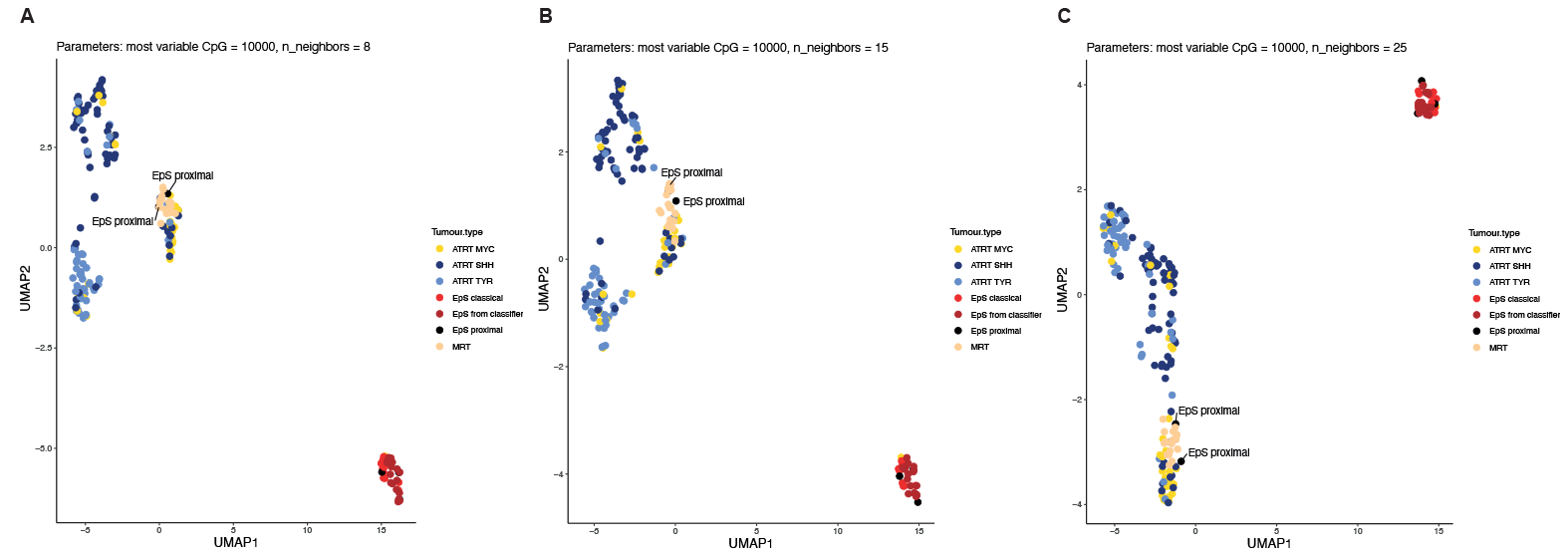
**

**Figure S5.** UMAP analysis including EpS (*n*= 16), EpS from the classifier (*n*= 18), MRT (*n*= 17), and ATRT (*n*= 150) samples with different n_neighbours values, *n*= 8 (A), *n*= 15 (B), and *n*= 25 (C), in the regression model.


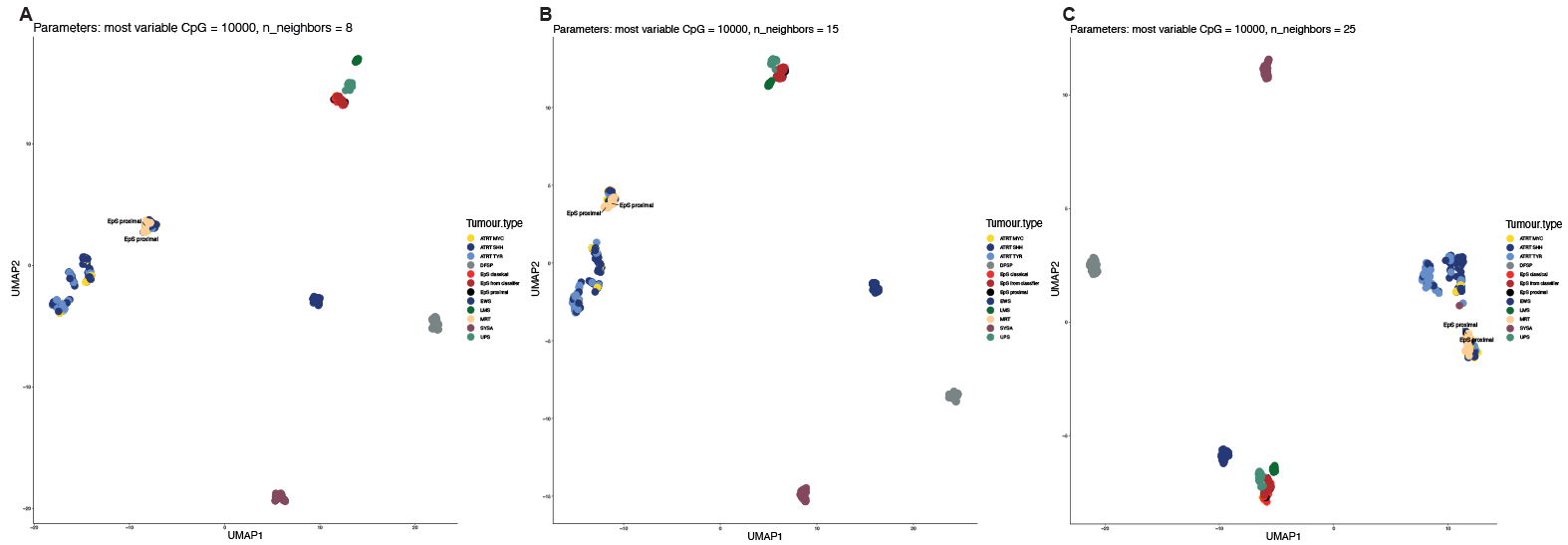


**Figure S6.** UMAP analysis including EpS (*n*= 16), MRT (*n*= 17), EpS from the classifier (*n*= 18), Ewing’s sarcoma (EWS, DKFZ) (*n*= 35), synovial sarcoma (SYSA, DKFZ) (*n*= 39), dermatofibrosarcoma protuberans (DFSP, DKFZ) (*n*= 37), class undifferentiated sarcoma (UPS, DKFZ) (*n*= 22), and leiomyosarcoma (LMS, DKFZ) (*n*= 16) with different n_neighbours values, *n*= 8 (A), *n*= 15 (B), and *n*= 25 (C), in the regression model.


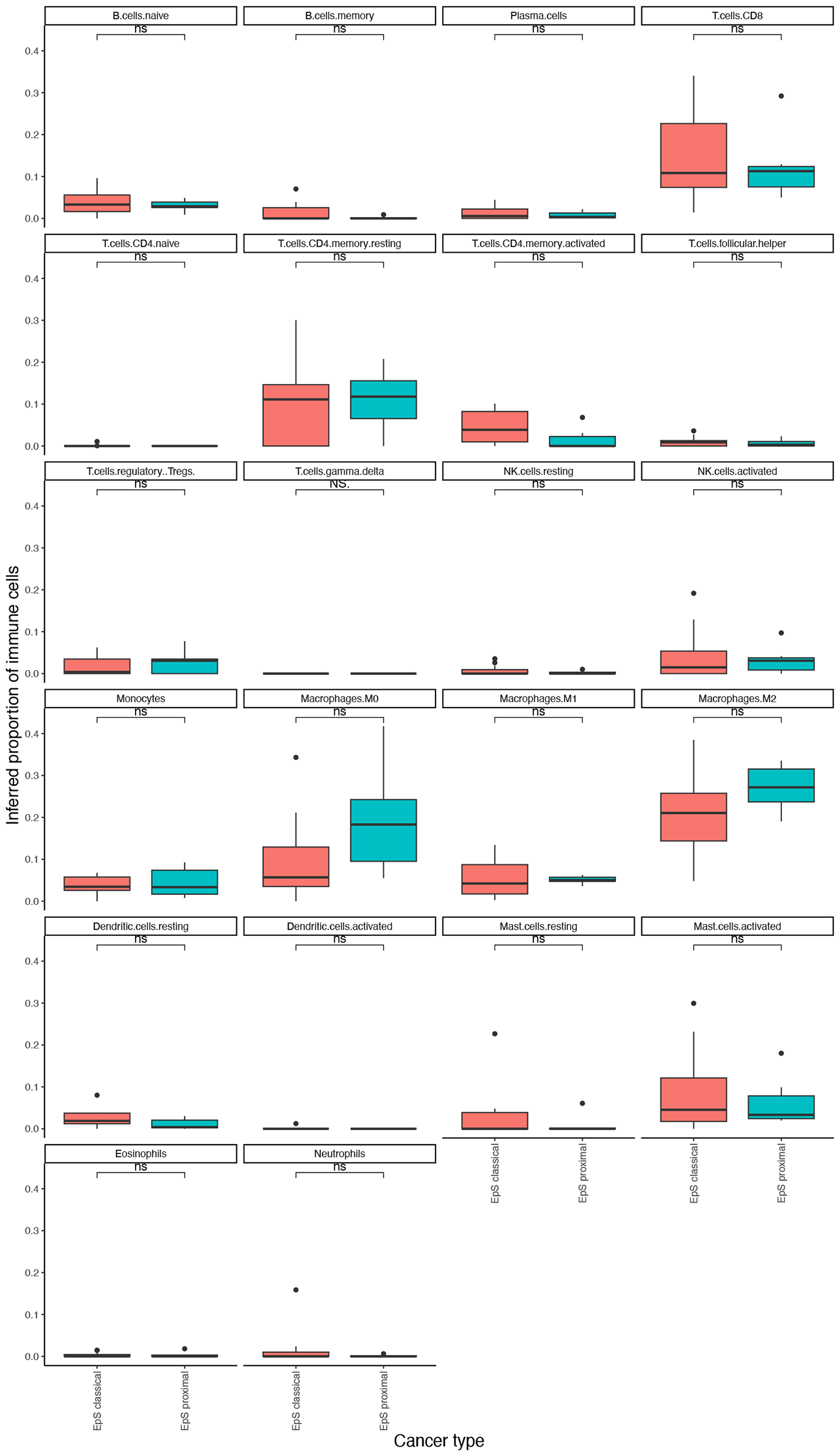


**Figure S7.** Boxplots comparing all inferred proportions of immune cells in the two EpS subtypes (*n*= 18). ns (not significant) p > 0.05, * p ≤ 0.05, ** p ≤ 0.01, *** p ≤ 0.001, **** p ≤ 0.0001.


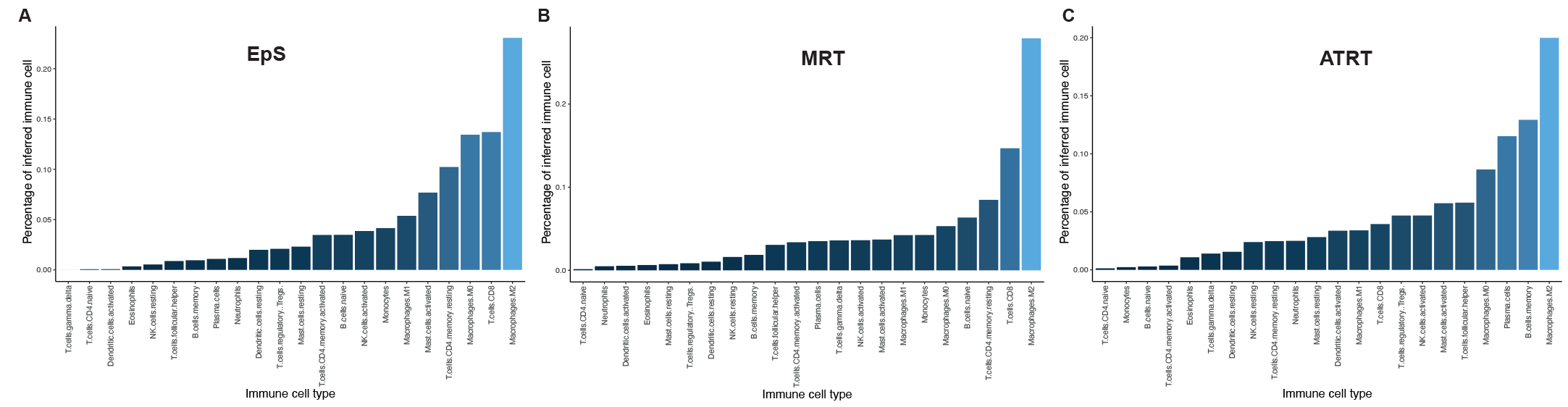


**Figure S8.** Barplots showing the inferred fraction of the 22 immune cell types included in the CIBERSORT matrix reference in EpS (*n*= 18) (A), MRT (*n*= 40) (B), and ATRT (*n*= 49) (C).


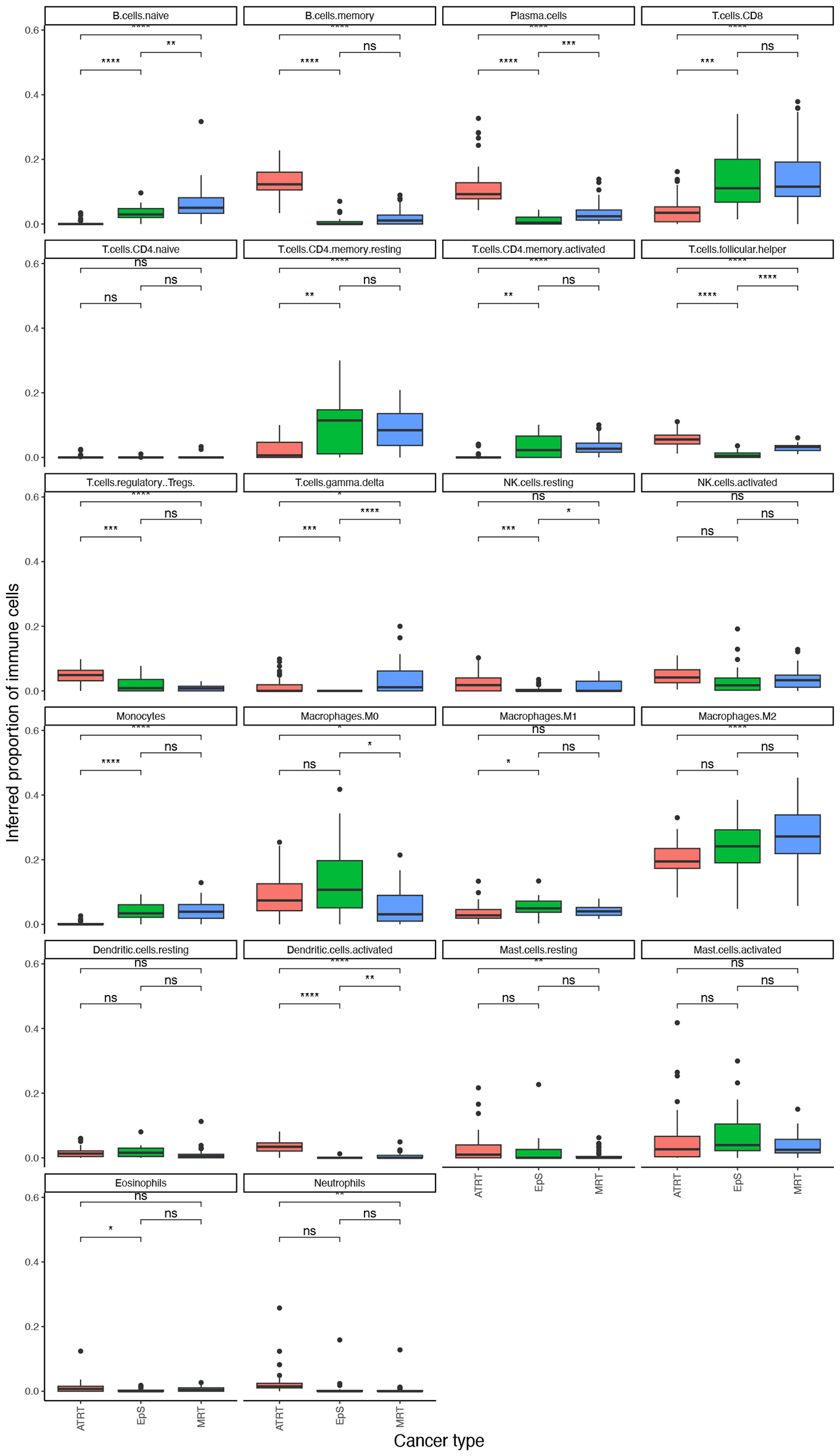


**Figure S9.** Boxplots comparing all inferred proportions of immune cells in *SMARCB1*-deficient neoplasms, EpS (*n*= 18), MRT (*n*= 40), and ATRT (*n*= 49). ns (not significant) p > 0.05, * p ≤ 0.05, ** p ≤ 0.01, *** p ≤ 0.001, **** p ≤ 0.0001.
